# Dose‐response association between step count and cardiovascular disease risk markers in middle‐aged adults

**DOI:** 10.1111/sms.14173

**Published:** 2022-04-28

**Authors:** Mark Hamer, Joanna M. Blodgett, Emmanuel Stamatakis

**Affiliations:** ^1^ Division Surgery Interventional Science Institute of Sport Exercise and Health University College London London UK; ^2^ Charles Perkins Centre School of Health Sciences Faculty of Medicine and Health The University of Sydney Sydney Australia

**Keywords:** cardiovascular disease, epidemiology, exercise, physical activity, population

## Abstract

Several step‐based daily targets have been widely circulated, but there is a lack of empirical population‐based evidence to support such guidance. We examined dose‐response associations between step count and classical CVD risk markers (glycated hemoglobin, high density lipoprotein cholesterol, triglycerides, and C‐reactive protein) in 4665 adults (aged 46 years; 51.4% female) in a cross‐sectional study. Step counts were measured from a thigh mounted accelerometer (activPAL) worn over 7 days. The shape of the dose‐response curve for most risk markers was “L‐shaped,” with linear risk reduction up to around 10 000 steps a day. Controlling for stepping intensity did not materially alter our results.

## 1 INTRODUCTION

The World Health Organization physical activity guidelines suggest people should engage in at least 150–300 min of moderate‐intensity physical activity or 75–150 min of vigorous‐intensity each week.^1^ However, daily step count has been used as a popular approach to provide relatively simpler physical activity targets for the general public. There has been limited research to empirically examine the shape of the dose‐response curve between step count and health outcomes which may contribute to the absence of formal recommendations on daily number of steps.^2^, ^3^, ^4^, ^5^, ^6^ A recent meta‐analysis of 10 cohort studies showed that the association between step count and mortality was L‐shaped, with 8.5% mean risk reduction every 1000 steps/day up to around 7500 steps/day.^2^ Another meta‐analysis showed that although the association between step count and cardiovascular disease (CVD) events was non‐linear, there continued to be positive benefits beyond 7500 steps.^6^ These data suggest that no minimum threshold exists for health benefits and some gains may be achieved beyond 7500 steps/day depending on the specific outcome.

The studies included in the meta‐analyses to date largely contained older adults, thus it is unclear if findings can be widely generalizable. As the absolute energy cost of walking and other daily activities is higher in older adults than younger adults,^7^ the health benefits of 7500 or any absolute volume of steps may vary by age and clinical outcomes. The aim of this study was to examine dose‐response associations between step count and CVD risk markers in middle‐aged adults.

## 2 METHODS

Data were drawn from the mid‐life, age 46, biomedical assessment of the 1970 British Cohort Study (BCS70) conducted in 2016–18^8^ utilizing a cross‐sectional design for the present analyses. Data collection comprised paper‐based self‐completion questionnaires, computer‐assisted personal interviewing, and nurse biomedical assessments during a home visit. All participants gave written informed consent and the study received ethical approval from the National Research Ethics Service (NRES) Committee South East Coast ‐ Brighton & Sussex (Ref 15/LO/1446).

Daily step counts were measured using a thigh‐worn accelerometer (activPAL3; PAL Technologies), worn continuously for 7 days according to the protocol previously described.^9^ Data were downloaded with PAL technologies software and analyzed using previously validated open‐access tools (java based ProcessingPAL algorithm).^10^ The first day of data were excluded, and subsequent days were defined as the 24 h between consecutive midnights. Only participants providing at least one valid day, defined as waking wear‐time more than 10 h per day, were included for further analysis. Non‐fasting blood samples were collected for the analysis of high density lipoprotein (HDL)‐cholesterol, triglycerides, glycated hemoglobin (HbA1C), and high sensitivity C‐reactive protein (hsCRP). All assays demonstrated acceptable reliability (CVs <5%).^8^ Covariates (all treated as categorical, except BMI) included sex, education, self‐rated health, disability, smoking, alcohol, and BMI.

In order to examine the shape of the dose‐response curve, we fitted restricted cubic spline models placing knots at the 10th, 50th, and 90th percentiles, as recommended by Harrell et al.^11^ and consistent with existing literature.^2^, ^6^ Consistent with previous studies,^2^ all cubic spline models were minimally adjusted for wear‐time and sex (no missing data); interactions with sex were assessed and if significant, results are presented separately for males and females. Non‐linearity between step count and each outcome was assessed using the Wald test. Linear regressions were used to estimate effect sizes of a 1000 step increase on risk markers, based on segments identified in the restricted cubic spline models. In sensitivity analyses, we additionally adjusted these models for covariates, including sex, education, self‐rated health, disability, smoking, alcohol, and BMI. In order to account for potential confounding effects of stepping intensity, our analyses were repeated after stratifying the sample by stepping intensity quartiles^4^ using time (min/d) spent above cadence of 100 steps per minute.^12^ We performed a further set of sensitivity analyses using 4 valid wear days as inclusion criterion (up to *n* = 4248, >90% of the main sample). All analyses were conducted using R statistical software (“rms” package).

## 3 RESULTS

The sample comprised of up to 4,665 participants with valid step count data and information on at least one biomarker (51.4% female). Average waking wear‐time was 15.9 ± 1.3 hours/day and 79.6% of the sample recorded at least 6 full days of wear. As previously reported,^9^ 11.8% of participants approached to take part declined to wear the device and were more likely to be male, smokers, report poorer health, and be obese. Daily step count in the included sample was normally distributed, ranging from 1128 – 32 352 (average 9532 ± 3653). There was low prevalence of self‐reported heart disease (2.2%), high blood pressure (8.6%), and diabetes (2.4%) within the sample. A description of the sample is provided in Table S1. Daily step count was related to sex (mean difference women [ref] vs. men; 233, 95% CI, 36 – 431), smoking (smokers [ref] vs. none‐smokers; 667, 396 – 937), self‐rated health (poor [ref] vs. excellent; 2314, 1649 – 2979), disability (severe [ref] vs. none; 1640, 1085 – 2195), education (none [ref] vs. degree; −325, −40 to −611), and body mass index (obese [ref] vs. none‐obese; 1190, 966 – 1415).

**FIGURE 1:**
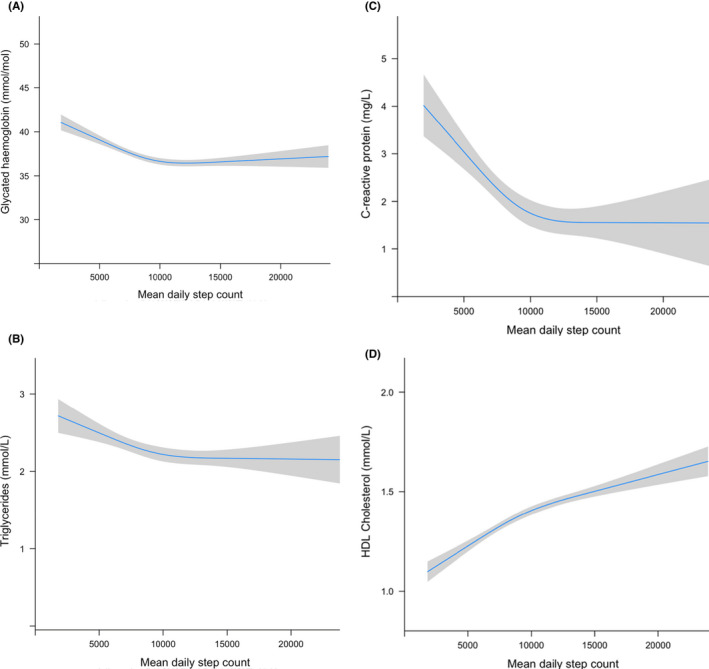
Restricted cubic spline models to examine association between step count and (A) Glycated hemoglobin, (B) Triglycerides, (C) C‐reactive protein, (D) HDL‐Cholesterol. Models were adjusted for wear‐time and sex. Grey border reflects 95% confidence interval around the mean

We observed consistent associations between daily step count and all CVD risk markers in wear‐time and sex‐adjusted spline models (Figure 1). The associations were non‐linear for all risk markers (Wald test: *p *< 0.05 for all), and no sex interactions were observed. For each 1000 steps, there was a linear inverse association with HbA1C of −0.58 (95% CI, −0.76, −0.41 mmol/mol; *n* = 4576) up to around 10 000 steps when the curve flattened (*p *= 0.69; Figure 1A). Similar linear associations were seen for triglycerides, per 1000 steps, (−0.04; −0.08, −0.01 mmol/L; *n* = 2678) and CRP (−0.23; −0.36, −0.10 mg/L; *n* = 2678) with curves flattening at around 10 000 steps (*p *= 0.59 and 0.99, respectively; Figure 1B,C). For each 1000 steps, there was a linear association with HDL‐C of 0.034 (0.026, 0.042 mmol/L; *n* = 4576) up to around 10 000 steps when the curve flattened but to a lesser extent than the other biomarkers [beyond ~10 000 steps, the increase per 1000 steps was 0.014 (0.007, 0.021) mmol/L]. (Figure 1D). In fully adjusted models, effect estimates were attenuated, albeit remained significant for HbA1C and HDL (see Table S2).

In sensitivity analyses stratified by stepping intensity, the associations of step count with HDL‐C and HbA1C were largely consistent across quartiles of stepping intensity (see Supplementary Figures). Owing to reduced sample size, we were unable to explore these analyses for CRP and triglycerides. In our sensitivity analyses using 4 valid wear days as inclusion criterion (instead of one), results were not materially changed.

## 4 DISCUSSION

There have been a variety of step‐based targets proposed,^13^, ^14^ but lacking in empirical evidence. We aimed to examine dose‐response associations between step count and classical CVD risk markers in middle‐aged adults. Our key findings suggest the shape of the dose‐response curve for most risk markers was L‐shaped, with linear associations up to around 10 000 steps a day. This is in contrast to previous studies on premature mortality that largely demonstrated optimal benefit at 7500 steps a day,^2^ albeit for CVD events there continued to be some positive benefits beyond this cut‐point.^6^ Nevertheless, these studies have been conducted in older adults. In one of the few general population studies with a relatively younger sample (mean age 56.8 years), the association between step count and mortality appeared more linear.^4^ Recent data have also confirmed a difference in plateau for the step count ‐ mortality curve in younger and older adults.^15^ Consistent with other studies,^3^, ^4^ taking measures to control for stepping intensity did not materially alter our results. Thus, associations between stepping and health appear to be primarily driven by volume, not intensity.

Several studies have previously explored associations between step count and cardiometabolic risk markers.^16^, ^17^, ^18^ However, many of these studies have comprised small, unrepresentative samples.^18^ Results have been inconsistent, particularly for dysglycemia outcomes where null findings were sometimes reported.^18^ Most studies did not attempt to examine the shape of the curve, although in those that did linearity was supported.

A key strength is the sample of healthy middle‐aged adults before the onset of major chronic disease, thus reducing the possibility of reverse causation in this cross‐sectional study. The distribution of the step count data allowed for an examination of dose‐response associations across the full range, even at higher levels that has been a limitation of cohorts containing older adults. Although the analyses were adjusted for key confounders, we cannot discount the likelihood of residual confounding. As is the case in most population studies, respondents that did not consent to wear a device tended to be less educated and report poorer health that may have introduced bias. Participants with greater compliance to wearing the device were also generally healthier although device wear did not appear to influence our results. Step count data were collected during a single week of the year and may be subject to seasonal fluctuations, although repeatability studies have demonstrated stability of step counts over 2–3 years.^19^ We chose to utilize a stepping intensity variable with a threshold of 100 steps/min, albeit this has been validated in the laboratory setting but not free‐living conditions.

Step count targets can be communicated in a way that is easily understood and memorized for the general public. Our findings suggest linear, beneficial associations between step count and CVD risk markers up to around 10 000 steps a day in middle‐aged adults.

## CONFLICT OF INTEREST

None of the authors declare any conflicts of interest.

## Supporting information

Supplementary Material

## Data Availability

The data that support the findings of this study are openly available in UK Data Service at https://beta.ukdataservice.ac.uk/datacatalogue/series/series?id=200001#!/access‐data, reference number 8547.
