# Supplementary material for: Dose‐response association between step count and cardiovascular disease risk markers in middle‐aged adults
Source: Scand J Med Sci Sports. 2022 Apr 28;32(7):1161–5. doi: 10.1111/sms.14173 (PMC9428935; doi:10.1111/sms.14173)

**Online supplementary material**

**Table S1.** Descriptive characteristics of the sample.

|  | **Females (n=2397)** | **Males (n=2268)** |
| --- | --- | --- |
| Glycated hemoglobin (mmol/mol), mean ±SD | 35.8 ±6.5 | 37.4 ± 8.3 |
| Triglycerides (mmol/L), median (Q1, Q3) | 1.2 (0.9, 1.7) | 1.8 (1.3, 2.7) |
| C-reactive protein (mg/L), median (Q1, Q3) | 1.0 (0.5, 2.6) | 1.0 (0.5, 2.2) |
| HDL cholesterol (mmol/L), mean ±SD | 1.68 ±0.44 | 1.37 ±0.38 |
| Daily step count, mean ±SD | 9702 ±3814 | 9371 ±3487 |
| Self-reported cardiometabolic diseases, n(%)  heart disease  high blood pressure  diabetes | 42 (1.8)  166 (6.9)  37 (1.5) | 59 (2.6)  233 (10.3)  74 (3.3) |
| Current smokers, n(%) | 415 (17.3) | 406 (17.9) |
| Alcohol consumption^a^. n(%)  Non-drinker  Non-problem drinker  Problem drinker | 251 (10.6)  1 741 (73.6)  373 (15.8) | 175 (7.8)  1 375 (61.4)  689 (30.7) |
| Self-reported health, n(%)  Poor  Fair  Good  Very good  Excellent | 92 (3.8)  282 (11.8)  586 (24.5)  930 (38.8)  507 (21.2) | 69 (3.0)  264 (11.6)  669 )29.5)  859 (37.9)  407 (18.0) |
| Disabled (EU- SILC^b^ classification), n(%) | 126 (5.3) | 82 (3.6) |
| Education, n(%)  None  Up to A levels or diploma  Degree or higher | 549 (23.2)  1 141 (48.1)  682 (28.8) | 649 (29.1)  969 (43.4)  613 (27.5) |
| BMI, mean ±SD | 27.8±5.8 | 28.4±4.5 |
| ^a^ Alcohol use disorders test- primary cary (AUDIT-PC)  ^b^ European Union Statistics on Income and Living Conditions, severely hampered | | |

**Table S2.** Fully adjusted models showing association between step count and biomarkers

|  | Model adjusted for sex + wear time (maximal sample) | Model adjusted for sex + wear time (complete cases) | Model adjusted for additional covariates ^a^ |
| --- | --- | --- | --- |
| HbA1c  ≤10 000 steps  >10 000 | N=4576  -0.58 (-0.76, -0.41), p<0.001  -0.02 (-0.11, 0.07), p=0.69 | N=4442  -0.57 (-0.75, -0.40), p<0.001  -0.02 (-0.11, 0.08), p=0.75 | N=4442  -0.22 (-0.39, -0.04), p=0.02  0.05 (-0.04, 0.14), p=0.28 |
| Triglycerides  ≤10 000 steps  >10 000 | N=2678  -0.04 (-0.08, -0.01), p=0.03  0.01 (-0.02, 0.04), p=0.59 | N=2592  -0.05 (-0.08, -0.01), p=0.02  -0.01 (-0.02, 0.04), p=0.40 | N=2592  -0.003 (-0.04, 0.04), p=0.90  0.03 (-0.002, 0.06), p=0.07 |
| CRP  ≤10 000 steps  >10 000 | N=2679  -0.23 (-0.36, -0.10), p<0.005  0.00 (-0.06, 0.06), p=0.99 | N=2593  -0.21 (-0.35, -0.08), p<0.005  0.00 (-0.06, 0.06), p=0.99 | N=2593  0.01 (-0.13, 0.15), p=0.88  0.03 (-0.03, 0.09), p=0.26 |
| HDL  ≤10 000 steps  >10 000 | N=4617  0.034 (0.026, 0.042), p<0.001  0.014 (0.007, 0.021), p<0.001 | N=4480  0.033 (0.025, 0.041), p<0.001  0.013 (0.006, 0.020), p<0.001 | N=4480  0.014 (0.006,0.022), p<0.001  0.006 (-0.001, 0.012), p=0.09 |

Estimates represent per 1,000 step increase separately for each segment of curve; P values reflect test for linearity using the Wald test.

^a^ Adjusted for sex, wear time, education, self-rated health, disability, smoking, alcohol, body mass index.

**Figure S1.** Association between step count and CVD risk markers stratified by stepping intensity (A lowest quartile; D highest quartile)

**HDL**


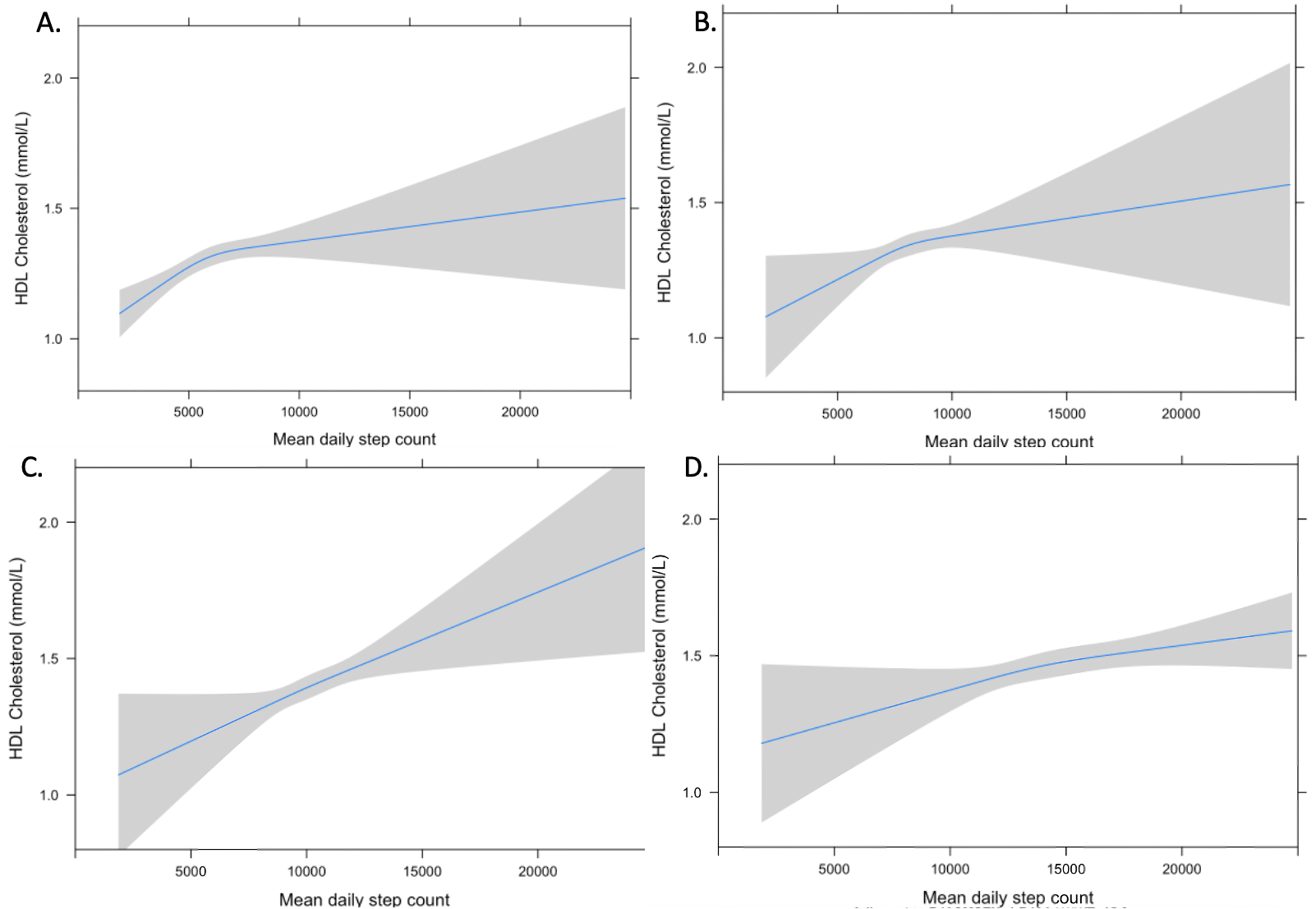


**HbA1c**


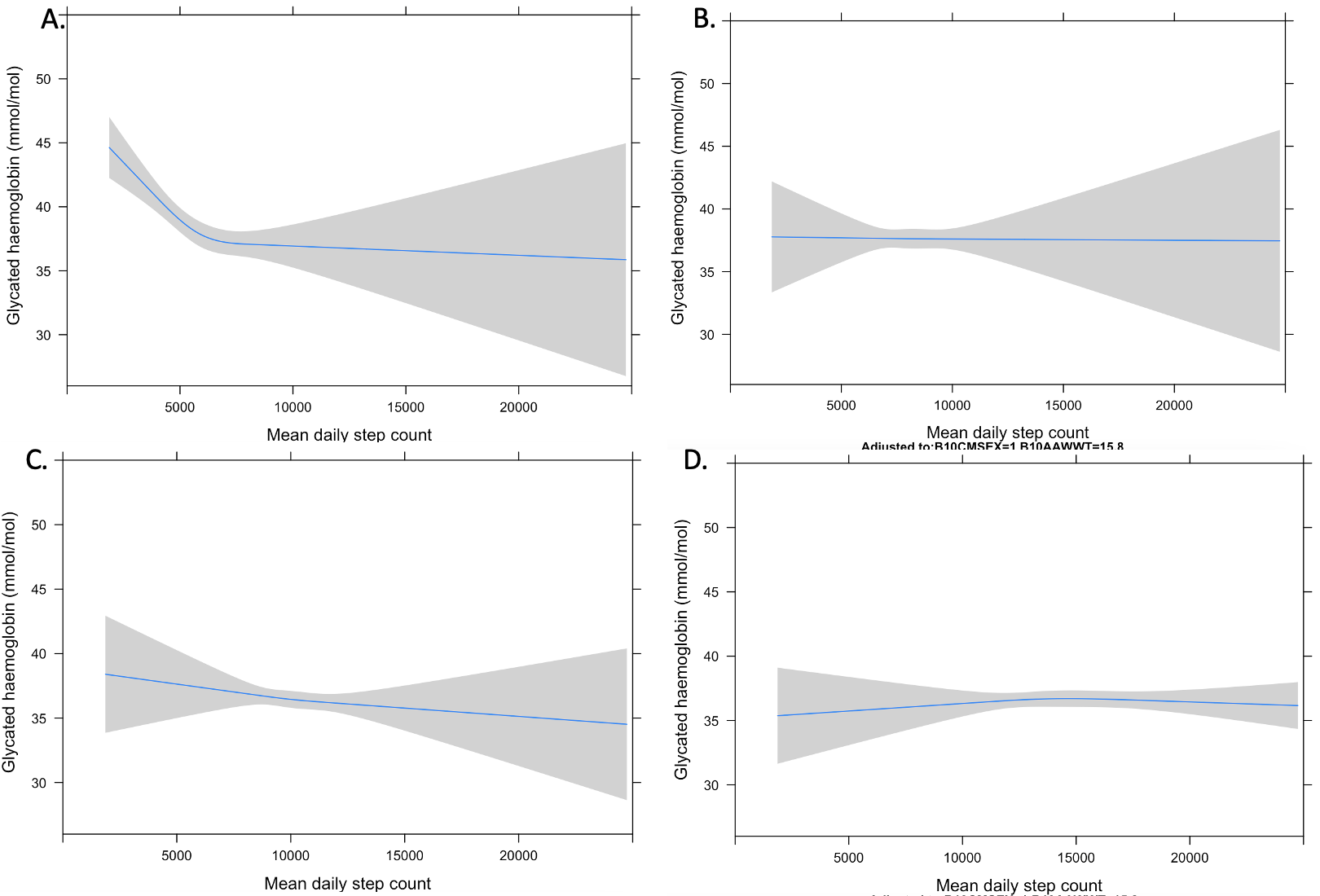

Supplement: Supplementary file 1 — Supplementary Material [file SMS-32-1161-s001.docx]
